# Correction: Biogenic sunflower oil-chitosan decorated fly ash nanocomposite film using white shrimp shell waste: Antibacterial and immunomodulatory potential

**DOI:** 10.1371/journal.pone.0307335

**Published:** 2024-07-12

**Authors:** Seham S. Alterary, Musarat Amina, Maha F. El-Tohamy

There are errors in the Funding statement. The correct Funding statement is as follows: The authors extend their appreciation to the Deputyship for Research & Innovation, Ministry of Education in Saudi Arabia for funding this research work through the project no. (IFKSURG1588). The funder has full participation in the manuscript beside financial support. The authors participated in visualization, formal analysis, and validation.
